# Integrative lncRNA landscape reveals lncRNA-coding gene networks in the secondary cell wall biosynthesis pathway of moso bamboo (*Phyllostachys edulis*)

**DOI:** 10.1186/s12864-021-07953-z

**Published:** 2021-09-04

**Authors:** Jiongliang Wang, Yinguang Hou, Yu Wang, Hansheng Zhao

**Affiliations:** Key Laboratory of National Forestry and Grassland Administration/Beijing for Bamboo & Rattan Science and Technology, Institute of Gene Science and Industrialization for Bamboo and Rattan Resources, International Centre for Bamboo and Rattan, 100102 Beijing, China

**Keywords:** LncRNA, Bamboo fast-growth, *Phyllostachys edulis*, Lignin, Secondary cell wall

## Abstract

**Background:**

LncRNAs are extensively involved in plant biological processes. However, the lack of a comprehensive lncRNA landscape in moso bamboo has hindered the molecular study of lncRNAs. Moreover, the role of lncRNAs in secondary cell wall (SCW) biosynthesis of moso bamboo is elusive.

**Results:**

For comprehensively identifying lncRNA throughout moso bamboo genome, we collected 231 RNA-Seq datasets, 1 Iso-Seq dataset, and 1 full-length cDNA dataset. We used a machine learning approach to improve the pipeline of lncRNA identification and functional annotation based on previous studies and identified 37,009 lncRNAs in moso bamboo. Then, we established a network of potential lncRNA-coding gene for SCW biosynthesis and identified SCW-related lncRNAs. We also proposed that a mechanism exists in bamboo to direct phenylpropanoid intermediates to lignin or flavonoids biosynthesis through the *PAL/4CL/C4H* genes. In addition, we identified 4 flavonoids and 1 lignin-preferred genes in the *PAL/4CL/C4H* gene families, which gained implications in molecular breeding.

**Conclusions:**

We provided a comprehensive landscape of lncRNAs in moso bamboo. Through analyses, we identified SCW-related lncRNAs and improved our understanding of lignin and flavonoids biosynthesis.

**Supplementary Information:**

The online version contains supplementary material available at 10.1186/s12864-021-07953-z.

## Background

Long non-coding RNAs (lncRNAs), defined as ncRNAs longer than 200 bp and could not translate into proteins, have attracted increasing attention. LncRNAs have both *cis*- and trans-regulatory functions [1]. LncRNAs can be produced in a sense or antisense direction from intergenic, intronic, or coding sequences of the genome. Depending on their location in the genome, they can be classified into different categories: long intergenic ncRNAs (lincRNAs), intronic ncRNAs (incRNAs), and natural antisense transcripts (NATs) [2]. LncRNAs can regulate gene expression at different levels through various mechanisms. They act by sequence complementarity or homology with RNA or DNA, and/or by structure, forming molecular frameworks and scaffolds for the assembly of macromolecular complexes [2]. In plants, the functions of lncRNAs in flowering regulation, reproductive developmental mediation, and stress response have been demonstrated [3, 4].

Advances in next-generation sequencing (NGS) technologies and computational methods have enabled researchers to *ab inito* identify novel lncRNAs *in silicon*. Genome-wide lncRNAs have been identified in many species, and made the first step toward a comprehensive and genome-scale perspective of lncRNA. For example, Matthew K Iyer et al. offered a transcriptome-based landscape of human lncRNAs [5]. In *Arabidopsis thaliana*, Xinyue Zhao et al. identified lncRNAs in the global genome [6]. In other plants, such as *cassava*, *Medicago truncatula*, and *Cucumis melo*, genome-wide lncRNAs had been identified [7–9]. Evolving lncRNA annotation profiles in multiple genomes contributed to the investigation of post-transcriptional regulation. In moso bamboo (*Phyllostachys edulis*), Taotao Wang et al. sequenced underground stem tissues and identified 1,989 lncRNAs [10]. However, this study could not provide a comprehensive map of lncRNAs, including highly tissue-specific lncRNAs, due to the limited tissues and datasets. Currently, RNA-seq datasets accumulated from different tissues or treatments of moso bamboo [11] could provide an opportunity to comprehensively identify lncRNAs in moso bamboo.

Secondary cell wall (SCW) is a key component of plant cell walls, and provided mechanic supporting for cells. The architecture and constitution of SCW affect the physical and mechanical properties of the wood resources [12]. In plants or *Saccharomyces*, lncRNAs are also involved in the regulation of SCW biosynthesis [13–15]. However, few SCW-related lncRNAs have been found in moso bamboo, which hinders the comprehensive understanding of SCW biosynthesis in moso bamboo. Here, we used a machine learning approach to refine a strategy of lncRNA identification and functional annotation based on the guidelines of previous studies [5, 6, 16, 17]. Then, we identified and annotated lncRNAs from additional datasets covering different tissues, different treatments, and different data types of moso bamboo. We also focused on SCW biosynthesis and excavated the SCW-related lncRNA-coding gene networks in moso bamboo.

## Results

### Genome-wide identification and functional annotation of lncRNAs

**Fig. 1:**
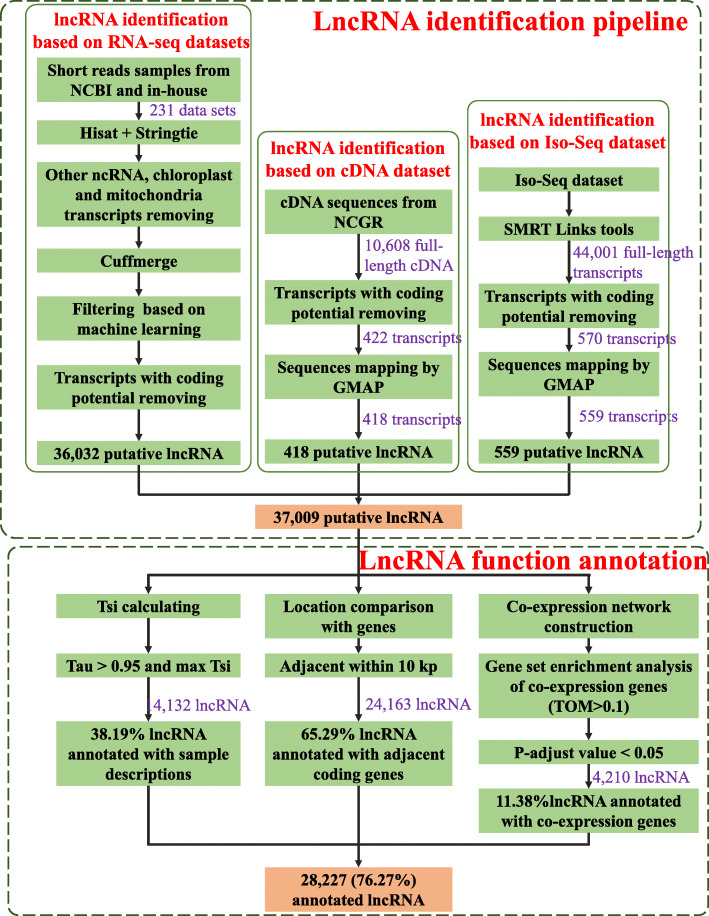
The pipeline of lncRNA identification and functional annotation. We identified 37,009 lncRNAs from RNA-Seq, cDNA and Iso-Seq datasets, and annotated 28,227 (76.27 %) lncRNAs’ function from 3 aspects, including space-time specialty, adjacent coding gene, and co-expression network

**Fig. 2:**
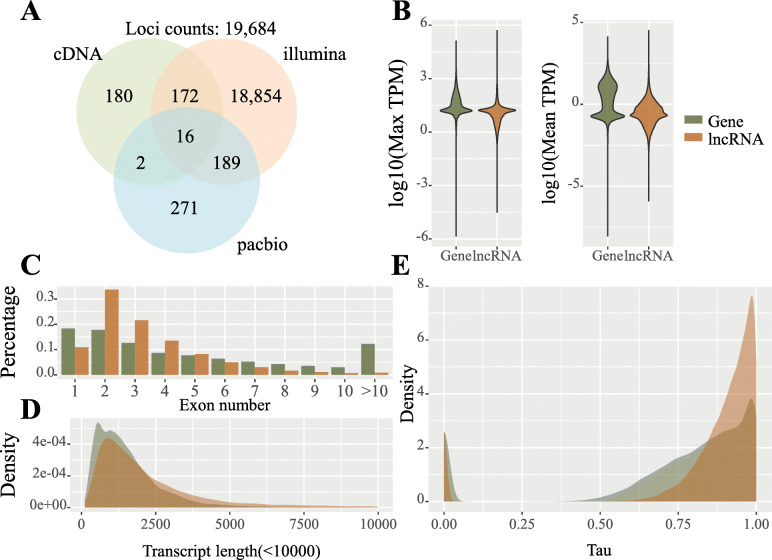
The Fig. of characters analysis of lncRNAs. **A** The Venn plot of loci of lncRNAs from different datasets. **B** TPM comparison between coding genes and lncRNAs. **C** Exon numbers comparison between coding genes and lncRNAs. **D** Transcript length comparison between coding genes and lncRNAs. **E** Tau comparison between coding genes and lncRNAs

We collected 231 RNA-Seq datasets, 1 Iso-Seq dataset, and 1 full-length cDNA dataset for comprehensive identification of lncRNAs in moso bamboo. The RNA-Seq datasets covered different tissues and multiple treatments of moso bamboo (Supplementary Table S1). Based on an improved strategy of machine learning methods (see Methods), we genome-widely identified lncRNAs in moso bamboo. The results showed that 14,610,124 transcripts were obtained after removing 2 low-mapping-rate samples in the assembly (Supplementary Table S2 and Fig. 1). After data preprocessing, we identified 37,009 potential lncRNAs, including 36,032 from RNA-Seq datasets, 418 from cDNA dataset, and 559 from Iso-Seq dataset (Fig. 1). The identified lncRNAs were distributed over 19,684 genomic loci, i.e., 16,348 lncRNAs were shared loci with other lncRNAs, accounting for ~ 44.2 % of the total lncRNAs (Fig. 1). The lncRNAs from the cDNA, Iso-Seq, and RNA-Seq datasets had 370, 478, and 19,231 loci, respectively. The Venn plot of lncRNAs from different dataset sources showed that the number of common loci among the three dataset sources was 16 (Fig. 2a). The low overlapped loci in lncRNAs from 3 dataset may due to the difference in sample size (RNASeq:231, Iso-Seq:1, cDNA:1) and the spatiotemporal specificity of samples. In addition, we characterized the lncRNAs in terms of TPM, exon number, length, and tissue-specific between coding genes and lncRNAs (Fig. 2b-e). For example, coding genes and lncRNAs showed similarity in terms of maximum TPM. In terms of exon number and transcript length, lncRNAs were close to coding genes. However, the average TPM of lncRNAs was lower than that of coding genes. In Tau, lncRNAs exhibited more tissue-specific members than coding genes, and the results were consistent with the characteristics of lncRNAs.

As with coding genes, the functional annotation of lncRNAs can guide researchers to study the function of the lncRNAs of interest. In the present study, we annotated lncRNAs using three strategies based on the previous study [17], i.e., tissue-specific analysis, adjacent coding gene analysis, and co-expression network analysis (see Methods). Based on the tissue-specific analysis, we annotated 14,132 lncRNAs as tissue-specific lncRNAs, covering 38.19 % of all lncRNAs (Fig. 1). For example, one lncRNA, TCONS_00006068, had a Tau value of 0.9964 and its maximum tsi value was 0.6564 in shoot tissue of the SRR6171236 dataset, so we identified this tissue description of SRR6171236 dataset, as an annotation of TCONS_00006068. Next, we identified the adjacent genes within 100 kb of the lncRNAs and annotated 65.29 % of the lncRNAs. Finally, we annotated 4,210 lncRNAs using the co-expression analysis and GSEA, covering 11.38 % of all lncRNAs. After statistical analysis, the functional annotation of 28,227 (76.27 %) lncRNAs was successfully predicted. By Venn diagram, we found that a total of 1,032 lncRNAs simultaneously annotated by all three methods (Supplementary Fig. S1). According to the functional annotation, the terms related to RNA, photosynthesis, terpenoid, and cell wall were mostly enriched (Supplementary Table S4). The functional annotations from the three aspects provided a landscape of lncRNA function for further analysis.

### Uncovering the relationship between lncRNAs and coding genes in SCW biosynthesis

**Fig. 3:**
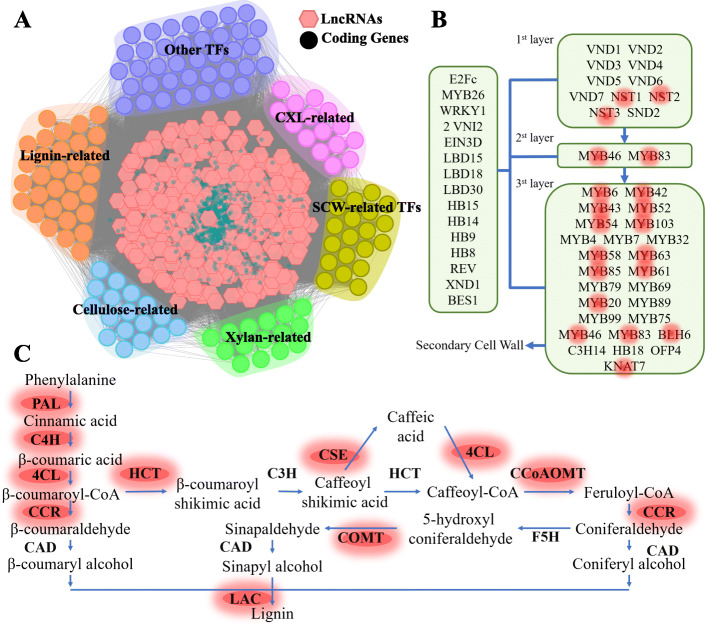
The potential regulating network of lncRNA-coding gene in SCW biosynthesis. **A** The co-expression network of lncRNA-coding gene in SCW formation. CXL means cellulose, xylan, and lignin. The other TFs in here belong to the same gene families in SCW-related TFs. **B,C** the regulation TFs in SCW formation and lignin biosynthesis pathway. The red light highlighted the genes found in **A**

We detected 315 SCW-related lncRNAs based on the lncRNA functional annotation (Supplementary Table S5). Among them, 44 lncRNAs were annotated as tissue-specific lncRNAs, mostly concentrated on shoots (Supplementary Table S6). For constructing a potential regulation network of the lncRNAs-coding genes, we extracted co-expression coding genes of these lncRNAs with a weight (TOM) > 0.1, and found a total of 1,668 coding genes with 176,393 pairs (Fig. 3).

The orthologs of these coding genes in *A. thaliana* and *O. sativa* were detected and their functions were annotated. Of the 1,668 coding genes, 98 were associated with SCW biosynthesis, including 34 lignin-related genes, 24 xylan-related genes, 31 cellulose-related genes, and 24 SCW-biosynthesis TFs (Supplementary Table S7-S10 and Fig. 3). The lignin-related genes and SCW-biosynthesis TFs could be mapped to the pathway of lignin biosynthesis [18] and the regulatory network of SCW biosynthesis [19], respectively (Fig. 3). In the regulatory network of SCW biosynthesis, 3, 2, and 13 TFs were mapped to the layers of 1st, 2st and 3st, respectively. We also identified the binding sites of SCW biosynthesis-related TFs in these lncRNAs (Supplementary Fig. S2). A total of 208 lncRNAs, accounting for 66 % of SCW-related lncRNAs, obtained one or more SCW-biosynthesis TF binding sites (Supplementary Table S11). We also detected another 38 TFs belonging to the gene families of SCW-biosynthesis TFs, such as *PH02Gene37942* (*OsMYB14*), *PH02Gene22729* (*OSH15*), and *PH02Gene06702* (*OsSND3*) (Supplementary Table S12). The lignin-related genes covered 70.1 % (12/17) of the enzyme gene families in the lignin biosynthesis pathway (Fig. 3). These results may indicate that lncRNAs have a strong influence on SCW biosynthesis through their involvement in regulating the biosynthesis of critical components in SCW, such as lignin, cellulose, and hemicellulose.

To investigate the expression profiles of SCW-related lncRNAs and genes in the co-expression network of moso bamboo shoots during the rapid growth phase, we constructed the expression heatmaps of these lncRNAs and genes, respectively, in shoots of different diameters. This dataset was released by Yongsheng Wang et al. in 2019. According to Supplementary Fig. S3A, most genes had higher expression profiles in the higher shoots. We then classified the genes into 12 clusters based on their expression patterns (Supplementary Fig. S3B). The genes in clusters 1–9 tended to have a moderate increase in expression in the lower shoots, a sharp increase of expression in the middle shoots, and then up to their stable high expression or a small decrease in the higher shoots. The gene expression in clusters 10–12 tended to increase sharply until 3 m tall shoots and then decreases sharply thereafter. Additionally, most lncRNAs were also highly expressed in the higher shoots (Supplementary Fig. S4). We also divided these lncRNAs into 12 clusters according to their expression patterns (Supplementary Fig. S4). As with the first gene group (gene clusters 1–9), the first group of lncRNAs (lncRNA clusters 1–4 and lncRNA clusters 6–10) showed a stable increase in expression in the lower shoots, a large increase in the middle shoots, and a stable or slight decrease in the higher shoots. LncRNA cluster 12 showed similarity to the second gene types (gene clusters 10–12), with a sharp increase in the expression of the lower shoots, a steady high expression of the middle shoots, and a sharp decrease in the expression of the higher shoots. LncRNA cluster 11 showed high expression in the lower shoots, sharply decreased, and increased in the middle shoots, and then reached high expression in the higher shoots. LncRNA cluster 5 showed high expression both in the lower shoots and in the higher shoots, low expression in the middle shoots. The result of the lncRNAs and genes can be further clustered to different groups, indicating the presence of some distinct and tightly coordinated clusters of genes and lncRNAs in the fast-growing shoots. The heatmap and expression pattern results can help us comprehend the network of lncRNAs-coding genes in the fast-growing shoots of moso bamboo.

**Fig. 4:**
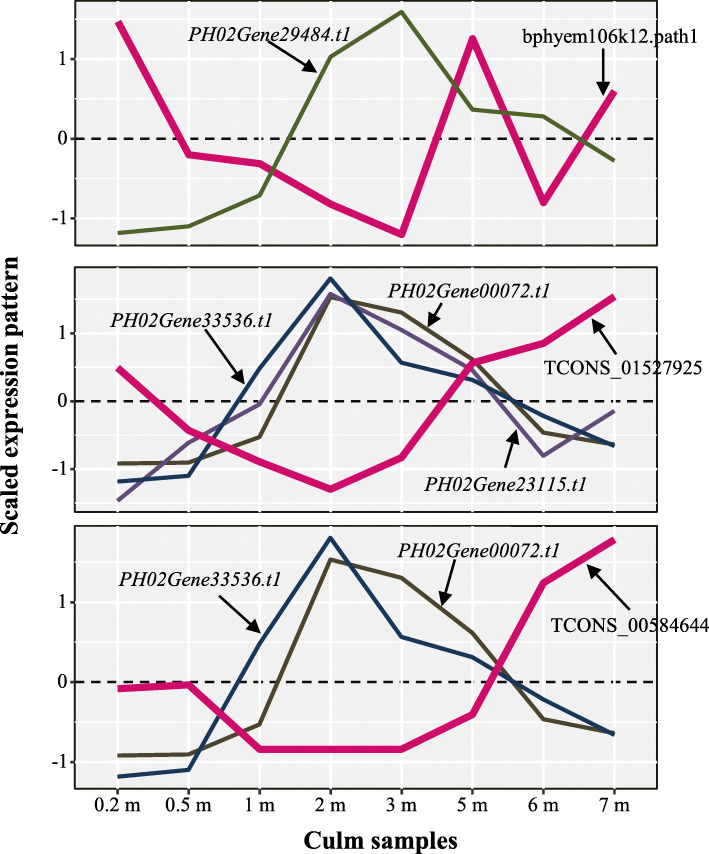
The negative correlation between SCW-related lncRNAs and coding genes. The mulberry lines showed the expression pattern of lncRNAs and others belong to coding genes. The scaled expression pattern of each object is the scaled TPM by scale() in the R script

To investigate the potential negative regulation of SCW biosynthesis in fast-growing shoots, we comprehensively scanned potential negative regulatory pairs of lncRNAs-encoding genes using expression pattern analysis and the dataset from Wang et al. in 2019 [20]. As a result, three lncRNAs, including bphyem106k12.path1, TCONS_01527925, and TCONS_00584644, were found to have potential negative regulatory functions in SCW biosynthesis (Fig. 4). Based on the scaled expression pattern using the scale function in R script, we detected 3 sets of negative expression patterns, i.e., (1) TCONS_01527925 vs. *PH02Gene33536*, *PH02Gene00072*, and *PH02Gene23115*; (2) TCONS_00584644 vs. *PH02Gene33536* and *PH02Gene00072*; (3) bphyem106k12.path1 vs. *PH02Gene29484*. The orthologs of all four genes in the above negative expression model were detected, i.e., the ortholog of *PH02Gene29484* was *Os01t0631100-01* (*Cas1p-like*) with e-value = 0; the ortholog of *PH02Gene33536* was *AT4G09990.1* (*GXM2*) with e-value < 5.34e-102; the ortholog of *PH02Gene00072* was *AT5G01360.1* (*TBL3*) with e-value < 3.03e-163; the ortholog of *PH02Gene23115* was *AT4G18990.1* (*XTH29*) with e-value < 1.26e-110. These orthologs were potential or confirmed SCW-related genes [21–24]. Thus, the results suggested that these three lncRNAs may exert their function by negatively regulating the SCW-related genes in the shoots.

### Identification of differentially expressed lncRNAs in a fast-growing model of moso bamboo shoots

**Fig. 5:**
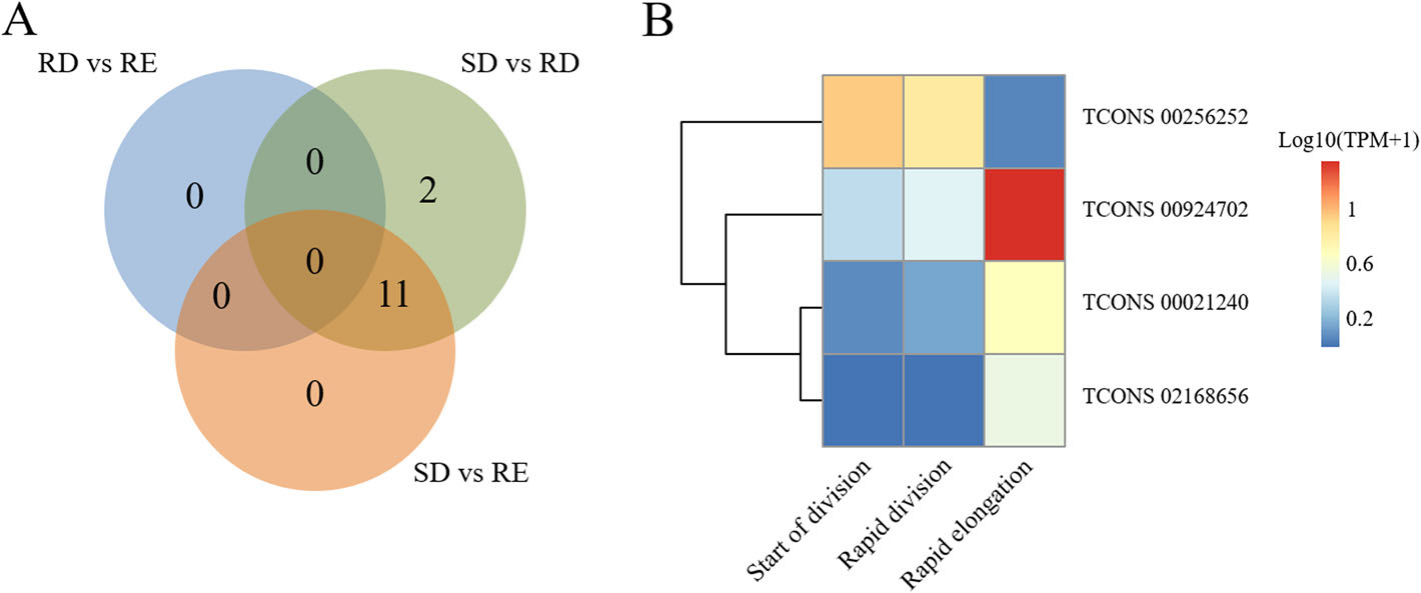
DELncRNAs analysis. **A** The Venn plot of DELncRNAs in SD, RD and RE stages. **B** The heatmap of 4 DELncRNAs

We compared the expression profiles of SCW-related lncRNAs based on the fast-growing model of moso bamboo shoots, with relevant data released by Gui-Yun Tao et al. in 2020 [25]. In the model, moso bamboo shoots were divided into three representative stages during rapid growth: start of division (SD), rapid division (RD), and rapid elongation (RE). Based on the analysis of DElncRNAs, we identified 11 DElncRNAs (Fig. 5A and Supplementary Table S13). According to the Venn diagram (Fig. 5A), there are no DElncRNAs between RD and RE, 11 DElncRNAs between SD and RD, and 13 DElncRNAs between SD and RE. GSEA results based on their co-expression showed 4 DElncRNAs earned significant GSEA results (p-adjust value < 0.005), including TCONS_00021240, TCONS_00256252, TCONS_00924702 and TCONS_02168656 (Supplementary Fig. S5). For example, TCONS_00021240 earned a large variety of GO terms about photosynthesis (GO:0019684, photosynthesis, light reaction; GO:0009767, photosynthetic electron transport chain; GO:0010206, photosystem II repair; GO:0009657, plastid organization). TCONS_02168656 earned rich GO terms about light and heat (GO:0009408, response to heat; GO:0009642, response to light intensity) and phytol (GO:0033306, phytol metabolic process). This may indicate both lncRNAs supported shoot development by performing their photosynthetic function, which was greatly in line with previous studies, in which photosynthesis-related genes were found to have their essential roles in fast-growing shoots [26, 27]. Cell wall biosynthesis-related GO terms (GO:0009832, plant-type cell wall biogenesis; GO:0009834, plant-type secondary cell wall biogenesis) were found in GSEA results of TCONS_00924702, which is in accordance with the needs of cell wall accumulation during shoot elongation. Cell cycle-related GO terms (GO:0000278, mitotic cell cycle; GO:0051301, cell division; GO:0000910, cytokinesis) were enriched in TCONS_00256252. Additionally, the heatmap (Fig. 5B) showed that TCONS_00256252 was more expressed in SD than in RE, and the other 3 DElincRNAs were more expressed in RE than in SD. The results are consistent with its high expression in SD, and may suggest it may be involved in cell proliferation during shoot development.

### Comparative analysis of lignin and flavonoids biosynthesis within the *PAL/C4H/4CL* gene families

**Fig. 6:**
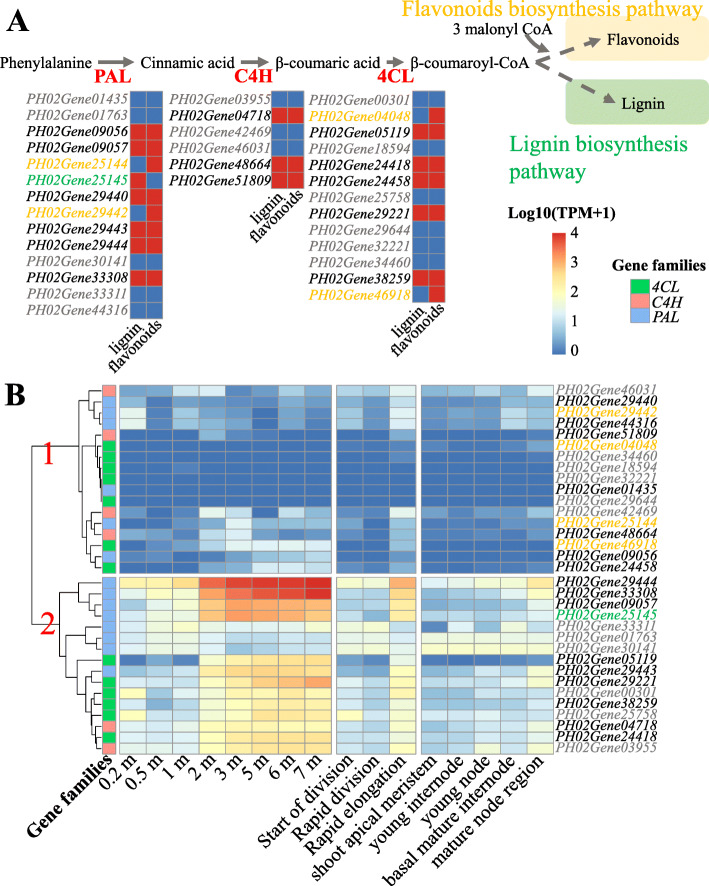
Comparison analysis between flavonoids and lignin biosynthesis with co-expression network. **A** The comparison results between flavonoids and lignin biosynthesis in *PAL*, *C4H*, and *4CL* genes. The red blocks showed the genes earned flavonoids or lignin biosynthesis -related co-expression genes. **B** The heatmap of *PAL, C4H* and *4CL* genes. The orange and green colors labeled names of genes are flavonoids and lignin preferred genes, respectively. The black labeled genes are the genes earning flavonoids and lignin biosynthesis-related co-expression genes. The grey labeled genes showed the genes without flavonoids and lignin biosynthesis-related co-expression genes

The lignin and flavonoids biosynthesis pathways have 3 significant common enzymes, i.e., 4CL, C4H, and PAL (Fig. 6) [28]. The comparative analysis of the lignin and flavonoids biosynthesis pathways in *PAL/C4H/4CL* genes will improve our understanding of these two pathways. Orthologous identification revealed 14 *PAL*, 6 *C4H*, and 13 *4CL* genes in moso bamboo (Supplementary Table S14). We then identified lignin- and flavonoid-related genes in the co-expression genes of these *PAL/C4H/4CL* genes using flavonoid and lignin-related GO terms. The results showed there were 18 *PAL/C4H/4CL* genes that contained flavonoids-related co-expression genes (Fig. 6A, Supplementary Table S15). Among them, 8 were *PAL* genes, 3 were *C4H* genes, and 7 were *4CL* genes. In contrast to flavonoids, 15 *PAL/C4H/4CL* genes contained lignin-related co-expression genes, of which, 7 were *PAL* genes, 3 were *C4H* genes, and 5 were *4CL* genes. Except for flavonoids- and lignin-related genes,14 *PAL/C4H/4CL* genes had no co-expression genes related to lignin or flavonoids. In addition, among the 17 flavonoids-related *PAL/C4H/4CL* genes, 4 genes (*PH02Gene25144*, *PH02Gene29442*, *PH02Gene04048*, and *PH02Gene46918*) were detected as co-expression genes that included only flavonoids-related genes, suggesting that these genes are flavonoid-preferred genes. Correspondingly, only 1 *PAL/C4H/4CL* gene (*PH02Gene25145*) was a lignin-preferred gene relative to flavonoids.

We also analyzed the expression of *PAL/C4H/4CL* genes in fast-growing shoots using the dataset released by the previous studies [25, 27] that reported the genes involving in the lignin biosynthesis pathway are active in the shoots, supporting the materials formation of shoot growth. According to the heatmap (Fig. 6B), the genes in *PAL/C4H/4CL* families could be divided into 2 groups, including the first group exhibited low expression in different shoots, whereas the second group showed high expression at some stages of shoots. This result may indicate a potential differentiation of *PAL/C4H/4CL* genes in moso bamboo shoot development. Furthermore, the flavonoid-preferred genes and lignin-preferred genes in *PAL/C4H/4CL* gene families belonged to the first and second groups, respectively. The results hinted an existence fundament of lignin- or flavonoid-preferred genes in the transcription level and supported the identification of preferred genes.

## Discussion

For comprehensively identifying lncRNA in moso bamboo, we collected 231 RNA-seq datasets, 1 Iso-Seq dataset, and 1 full-length cDNA dataset. These datasets covered different tissues, and distinct treatments of moso bamboo (Supplementary Table S1), and provided an unprecedented opportunity to identify genome-wide lncRNAs. We use machine learning approaches to refine the lncRNA identification and functional annotation pipeline from to the previous researches [5, 6, 16, 17] and provided a comprehensive lncRNA map of moso bamboo, which includes 37,009 lncRNAs. Nevertheless, the available RNA datasets in moso bamboo are much lesser compared to the model species. As of March 3, 2021, the SRA database (https://www.ncbi.nlm.nih.gov/sra/) holds a total of 1,012,204 RNA datasets of humans, 41,992 RNA datasets of *A. thaliana*, 14,253 RNA datasets of rice, but the ones in moso bamboo are 258. Considering the tissue-specific lncRNAs and the deficiency of moso bamboo RNA datasets, the current lncRNA map cannot fully cover potential lncRNAs. More transcriptome datasets of moso bamboo will be released as more transcriptome analyses and the Genome Atlas of Bamboo and Rattan (GABR) project [29] are carried out. Then, the developed lncRNA identification and functional annotation strategies will identify more lncRNAs to form a complete lncRNA map. We annotated the function of lncRNAs from 3 aspects. However, only ~ 65 % of samples were used to annotate tissue-specific lncRNAs due to inadequate sample descriptions (Supplementary Table S3), which hinders the functional annotation of lncRNAs. Here, we appeal to researchers that they should complete essential descriptions of samples whenever possible, which will provide great assistance for lncRNA functional annotation.

There is growing evidence that lncRNAs are essential regulators of cell wall formation. For example, some lncRNAs can regulate tomato (*Solanum lycopersicum*) fruit cracking by coordinating gene expression via the hormone-redox-cell wall network [13]. In *Saccharomyces cerevisiae*, lncRNAs are extensively involved in cell wall regulation [14]. The lncRNAs of antisense transcripts of *HvCesA6* produce small interfering RNAs to regulate cell wall biosynthesis in *Hordeum vulgare *[15]. Here, we identified 315 lncRNAs associated with SCW biosynthesis in moso bamboo based on the functional annotation of lncRNAs (Supplementary Table S5). These lncRNAs have co-expression relationships with TFs involved in SCW biosynthesis regulation network and the genes involved in lignin/cellulose/hemicellulose biosynthesis (Supplementary Table S7-10). In addition to the TFs listed in the SCW biosynthesis regulatory network [30], there are some other TFs that are co-expressed with these lncRNAs, including *PH02Gene37942* (*OsMYB14*), *PH02Gene22729* (*OSH15*), *PH02Gene06702* (*OsSND3*) (Supplementary Table S12). Previous studies have shown that *OsSND3* and *OsMYB14* are co-expressed with SCW-related genes in rice [30, 31]. *TWI1* is an essential factor limiting the movement of *OSH15*, which may enable proper programming of cell specification and promote lignin synthesis [32].

In addition, *AT4G09990.1* (*GXM2*) is involved in xylan synthesis in *A. thaliana*, and *SND1* regulates its expression [21]. *AT5G01360.1* (*TBL3*) is required for 3-O-Monoacetylation of xylan [22]. XTH is a class of xyloglucan-based substrate enzymes that catalyze xyloglucan [23]. *Os01t0631100-01* (*Cas1p-like*) is a homolog of *Cas1p*, and a mutation of a *Cas1p* member in *A. thaliana* caused decreased levels of acetylated cell wall polymers [24]. In the present study, three SCW biosynthesis-related lncRNAs with expression patterns opposite to the 4 genes mentioned above may be involved in cell wall biosynthesis or hemicellulose biosynthesis (Fig. 4). Therefore, the lncRNAs in the lncRNA-coding gene network may play an essential role by coordinating with SCW biosynthesis TFs or the genes involved in lignin, cellulose, and hemicellulose biosynthesis. Cell wall biosynthesis supports the development of bamboo shoot [25, 27]. Most SCW-related lncRNAs and their co-expressed genes show increased expression in low to middle shoots and slightly reduced or stable expression in high shoots, suggesting their function stages and patterns in fast-growing shoots. The fast-growing is the essential character of bamboo species and attracted attention of researchers [20, 25, 27, 33–36]. The identification of the lncRNAs that may play their indispensable roles in fast-growing stages of bamboo offered a new perspective of bamboo fast-growing to analysis.

The lignin content of moso bamboo is approximately 25 % of dry weight [37], which is higher than most herbaceous plants [38], and exhibits remarkable adaptations in lignin production. This may be due to the active nature of the lignin biosynthesis pathway during growth [25, 27]. However, the lignin biosynthesis pathway has a competing pathway, the flavonoids biosynthesis pathway, and both pathways contain three common enzymes (PAL, 4CL, and C4H) [28]. In some plants, PAL and 4CL enzymes are thought to have the ability to guide bio-metabolism to different pathways to regulate the biosynthesis of various compounds [39–41]. For example, apple (*Malus×domestica Borkh.*) can regulate the redistribution of phenylpropanoid intermediates to the flavonoid pathways while reducing the biosynthesis of lignin [42]. According to a study of *At4CL1* and *At4CL2* genes in *A. thaliana* and *Gm4CL4* gene in soybean (*Glycine max*), Santosh G. Lavhale et al. showed that some *4CL* genes are suitable for both lignin and flavonoid biosynthesis pathway in plants, and other *4CL* genes prefer only one of the lignin and flavonoids biosynthesis pathway [43]. Here, we identified 4 flavonoid-preferred and 1 lignin-preferred *PAL/4CL/C4H* genes in moso bamboo (Fig. 6). These results may indicate that, like apples, *A. thaliana*, soybeans, and other species, moso bamboo can direct phenylpropanoid intermediates specifically to the lignin or flavonoids biosynthesis pathway through members of the *PAL/4CL/C4H* genes. Additionally, identifying genes with a preference for flavonoid or lignin biosynthesis pathways could provide a possible starting point for shifting bio-metabolic intermediates to flavonoids or lignin biosynthesis pathways. It could be directed to alter lignin or flavonoids biosynthesis, increase lignin or flavonoids production, or change the composition of SCW to improve moso bamboo properties. This is of great significance to moso bamboo because of its dual use as an edible and material.

In addition, our results comparing the lignin and flavonoids biosynthesis pathways of *PAL/C4H/4CL* genes showed that metabolic intermediates may prefer the flavonoids biosynthesis pathway because there are more flavonoids-preferred genes of *PAL/C4H/4CL* than the lignin biosynthesis pathway. This sounds in conflict with the remarkable adaptations of bamboo in lignin production. However, the heatmap of *PAL/C4H/4CL* genes in fast-growing shoots showed flavonoids-preferred genes for *PAL/C4H/4CL* are relatively inactive. This may indicate that the remarkable adaptation of bamboo in lignin production is not based on the whole metabolic process of lignin, but only on some critical and dominant stages. But this hypothesis does not consider the potential diversity in period length and efficiency of *PAL/C4H/4CL* genes related to lignin or flavonoids biosynthesis, or differences in substrate content during metabolism.

## Conclusions

A comprehensive lncRNA map from the datasets covering multi tissues and treatments would promote the processing of lncRNA functional analysis and researches. Here, we collected multi datasets from distinct tissues and treatments and developed a pipeline of lncRNA identification and functional annotation to provide a comprehensive landscape of lncRNA in moso bamboo. The lncRNA map earns 37,009 members, and we annotated more than 65 % lncRNAs’ function. Next, we constructed a network of lncRNAs-coding genes of SCW biosynthesis and explored its potential functional pattern in fast-growing shoots through expression profile digging. Meanwhile, we compared flavonoids and lignin biosynthesis pathways through co-expression analysis of *PAL*, *4CL*, and *C4H* genes and suggested moso bamboo may have the ability of orienting phenylpropanoid intermediates to lignin or flavonoids biosynthesis pathway specifically through *PAL/4CL/C4H* genes. Furthermore, we identified 1 lignin-preferred and 4 flavonoids-preferred genes in *PAL/4CL/C4H* gene families, which may give a potential that controls phenylpropanoid intermediates into flavonoids or lignin biosynthesis pathway directedly.

## Methods and materials

### Datasets collecting and processing

For comprehensively identifying lncRNA candidates, we downloaded 231 RNA-Seq datasets from moso bamboo in NCBI (Supplementary Table S1). All RNA-Seq datasets were analyzed by FastQC v0.11.6 (http://www.bioinformatics.babraham.ac.uk/projects/fastqc/) with default parameters for quality statistics summary. Adapters and low-quality sequences were removed using Trimmomatic v0.36 [44] with the following parameters: LEADING:3, TRAILING:3, SLIDINGWINDOWS 4:15, MINLEN:50, and TOPPHRED64. In the data mapping, clean data were mapped to the moso bamboo genome [45] using HISAT2 v2.1.0 [46], with the following modifications from the default parameters: –min-intronlen 20, –max-intronlen 4000, and –rna-strandness RF. We removed two datasets because of their low mapping ratios (Supplementary Table S2). Then, we applied the default parameters of StringTie v1.3.5 [47] to assembly the transcripts. During transcript assembly, the transcripts Per Kilobase of exon model per Million mapped reads (TPM) values were obtained using StringTie.

In addition, we downloaded a cDNA dataset, which was released in 2010 from Moso Bamboo cDNA database (http://server.ncgr.ac.cn/mbcd/) [48]. We also collected the full-length transcripts from the previous study, which was assembled by using the single-molecule real-time isoform sequencing (Iso-Seq) dataset [45].

### Genome-wide identification of lncRNAs

We genome-wide identified lncRNA candidates of moso bamboo using the three datasets from RNA-Seq, Iso-Seq, and cDNA based on the guidelines of previous studies [5, 6, 16]. We provided a pipeline of identification and functional annotation process in Fig. 1. In RNA-Seq datasets, we applied BLAST + v2.9.0+ [49] and Cuffmerge v2.2.1 [50] to remove potential chloroplast, mitochondria, and other ncRNA sequences. We removed the transcripts with > 0.75 overlap ratio with chloroplast or mitochondria genomic sequences. For removing other ncRNA sequences, transcripts that overlapped with other ncRNAs > 0.3 were filtered. Then, Cuffmerge was applied to merge transcripts from different samples. For removing contaminating sequences, we treat transcripts with Cuffmerge classcode “i” as contamination according to a previous study [5] and developed a machine learning strategy to remove potentially contaminating transcripts. Briefly, we applied the Randomforest package v4.6-14 [51] in R and libsvm v3.24 [52] software with default parameters under five elements, including recurrence ratio, max TPM, mean TPM, transcript length, and exon counts, to classify two types of transcripts which are the transcripts with classcode “=” and “i”. Then we removed the transcripts identified as “i” in the machine learning step. Next, we reserved the transcripts with four Cuffmerge classcodes, including “j”, “u”, “x”, and “o”. Finally, we used domain filtering, sequence similarity filtering, length filtering, and coding ability filtering to remove the transcripts with the coding ability. In sequence similarity filtering, we used Swiss-Prot as a database and applied BLASTx under the parameters: e-value < 10 − 4, alignment length ≥ 40 aa, and percentage identity ≥ 35 %, to remove the transcripts with coding ability. In length filtering, the transcripts with length < 150 were removed. In coding ability filtering, we removed the transcripts with label is “coding” produced from CPC2 [53]. In domain filtering, we removed the transcripts with domain with e-value < 10 − 4 using pfamcan.pl (ftp://ftp.ebi.ac.uk/pub/databases/Pfam/Tools/). Furthermore, in identifying lncRNA from cDNA and full-length transcripts from the Iso-Seq dataset, we used the above strategy to remove transcripts with coding ability. We then mapped the candidate lncRNAs to the genome using GMAP v2017-11-15 [54] with default parameters.

### Functional annotation of LncRNAs from three aspects

We annotated the functions of candidate lncRNAs under the guideline of Chen, X. et al. [17]. The functional annotation included three aspects, i.e., tissue-specific analysis, adjacent coding gene analysis, and co-expression network analysis. In the tissue-specific analysis, we calculated the Tau value for each lncRNA based on tspex (https://tspex.lge.ibi.unicamp.br/) [55]. Some samples were removed because of the deficiency of description, resulting in 148 reserved samples (Supplementary Table S3). The lncRNAs with Tau > 0.95 was identified as a tissue-specific lncRNA, while the sample with maximum tissue specificity index (TSI) was identified as lncRNA-specific samples. Thus, sample-specific descriptions were used to annotate the corresponding lncRNAs. In the adjacent coding gene analysis, lncRNAs were annotated by considering the nearest gene (< 100 kb) of lncRNA as adjacent genes, based on the theory that lncRNAs may apply their functions by affecting closed genes. Co-expression network analysis is an effective strategy for lncRNA functional annotation. We used WGCNA package v1.69 [56] to conduct co-expression network analysis of lncRNAs and genes. Co-expression pairs with TOM > 0.1 were identified as co-expression networks. Then, gene set enrichment analysis (GSEA) was performed to annotate lncRNAs using each lncRNA’s co-expression coding genes. The GSEA was conducted by clusterprofiler v3.12 [57] with default parameters.

### Identification of TFs binding sites and orthologs

For identifying binding sites for transcript factors (TFs), we extracted the 3-kb upstream region of lncRNAs as promoters. TFs binding motif information was downloaded from PlantTFDB [58] (http://planttfdb.gao-lab.org/). Then, we used the subprogram fimo of MEME Suite v5.3.3 [59] to identify potential binding sites in lncRNA promoters with the parameters: --verbosity 1 --thresh 1.0E-6. Additionally, we used reciprocal best hit (RBH) BLAST [60] to identify potential orthologs between moso bamboo and *A. thaliana*, moso bamboo and *Oryza sativa*. We downloaded the protein sequences of *O. sativa* and *A. thaliana* from RAP-DB [61] (https://rapdb.dna.affrc.go.jp/download/irgsp1.html) and TAIR [62] (https://www.arabidopsis.org/), respectively. The top three hits of each RBH were identified as the best orthologous pairs. The pairs of e-values less than the peak of the e-value distribution of all the best hits were identified as the secondary orthologous pairs in orthologs identification.

### Differentially expressed lncRNAs analysis

We using limma v3.40.6 with default parameters [63] to conduct differentially expressed lncRNAs (DElncRNAs) analysis. The criteria of DElncRNAs are logFC > 2 and adj.P.Val < 0.001.

### Comparative analysis of lignin and flavonoids biosynthesis

We identified lignin- and flavonoid-related genes in the co-expression genes of *PAL/C4H/4CL* genes using flavonoid-related GO terms (GO:0009812: flavonoid metabolic process, GO:0009813: flavonoid biosynthetic process, GO:0009962: regulation of flavonoid biosynthetic process, GO:0009963: positive regulation of flavonoid biosynthetic process, GO:0009964: negative regulation of flavonoid biosynthetic process, GO:1,903,415: flavonoid transport from endoplasmic reticulum to plant-type vacuole) and lignin-related GO terms (GO:0009808: lignin metabolic process, GO:0009809: lignin biosynthetic process, GO:0046274: lignin catabolic process, GO:1,901,141: regulation of lignin biosynthetic process). Then, we compared these two pathways based on lignin- and flavonoid-related co-expression genes of *PAL/C4H/4CL* genes.

## Supplementary Information

Below is the link to the electronic supplementary material.

**Additional file 1. **

## Data Availability

The data-sets analysed during the current study are available in the SRA database (https://www.ncbi.nlm.nih.gov/sra) of NCBI repository. The SRA accession numbers can be found in Supplementary Table S1.

## Abbreviations

DElncRNAs: Differentially expressed lncRNAs
GABR project: Genome Atlas of Bamboo and Rattan project
GSEA: Gene set enrichment analysis
incRNAs: Intronic ncRNAs
Iso-Seq: Single-molecule real-time isoform sequencing
lincRNAs: Long intergenic ncRNAs
lncRNAs: Long non-coding RNAs
NATs: Natural antisense transcripts
NGS: Next-generation sequencing
RBH BLAST: Reciprocal best hit BLAST
RD: Rapid division
RE: Rapid elongation
SCW: Secondary cell wall
SD: Start of division
TFs: Transcript factors
TSI: Tissue specificity index
