# Supplementary material for: Integrative lncRNA landscape reveals lncRNA-coding gene networks in the secondary cell wall biosynthesis pathway of moso bamboo (Phyllostachys edulis)
Source: BMC Genomics. 2021 Sep 4;22:638. doi: 10.1186/s12864-021-07953-z (PMC8417995; doi:10.1186/s12864-021-07953-z)
Supplement: Supplementary file 1 — Additional file 1. [file 12864_2021_7953_MOESM1_ESM.zip › revised supplementary material figures, figures legends and table legends.docx]

## Supplementary Figures:

**Supplementary Fig. S1**


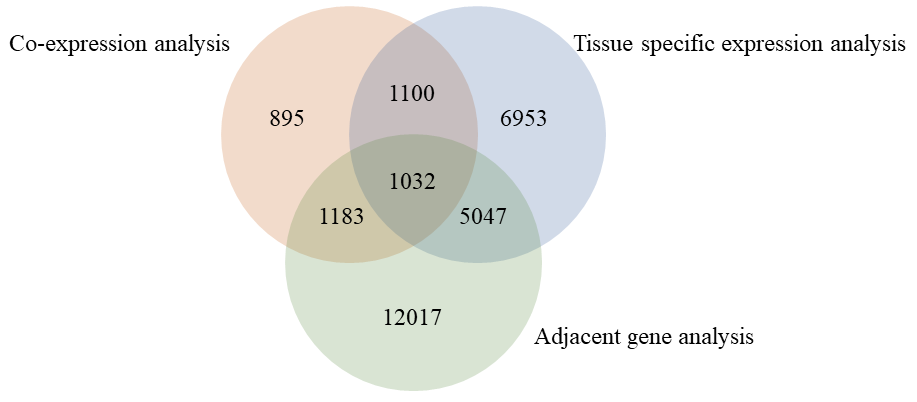


**Supplementary Fig. S1.** The Venn plot of lncRNA functional annotation summary.

The functions of lncRNAs were annotated using 3 strategies, including co-expression analysis, tissue-specific expression analysis and adjacent gene analysis. The number of lncRNAs annotated simultaneously by the 3 strategies was 1,032.

**Supplementary Fig. S2**


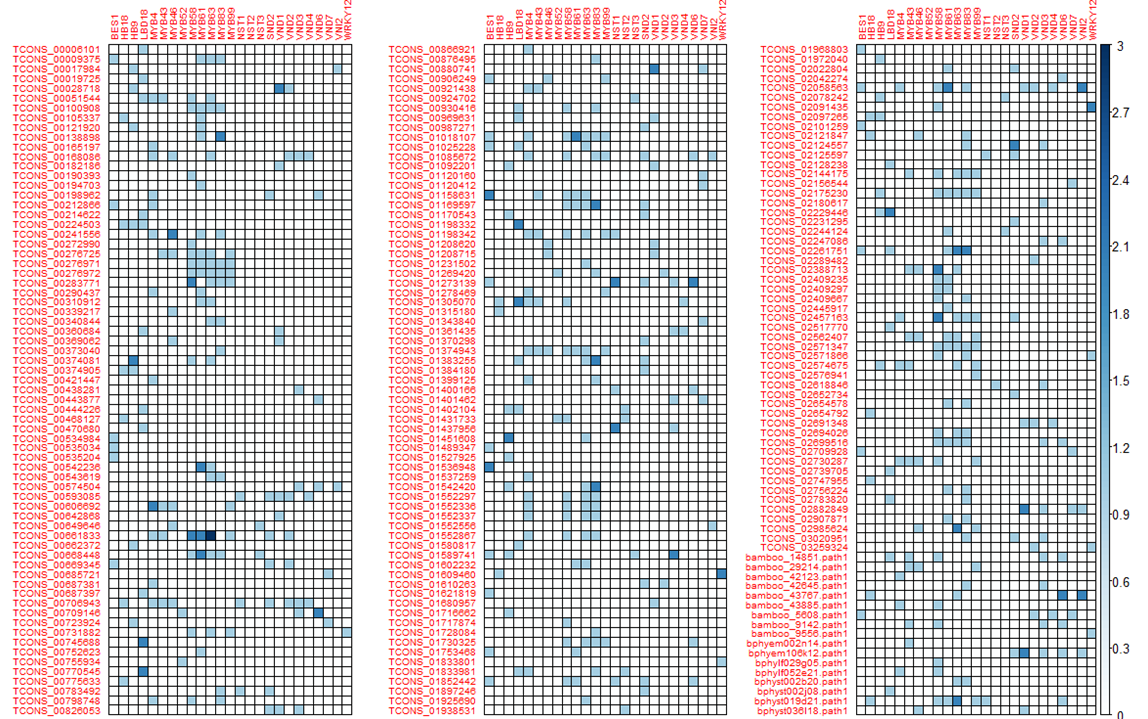


**Supplementary Fig. S2.** The binding sites of SCW-biosynthesis TFs in SCW-related lncRNAs.

The names of lncRNAs (left) and binding site of TF (top) were highlighted in red.

**Supplementary Fig. S3**


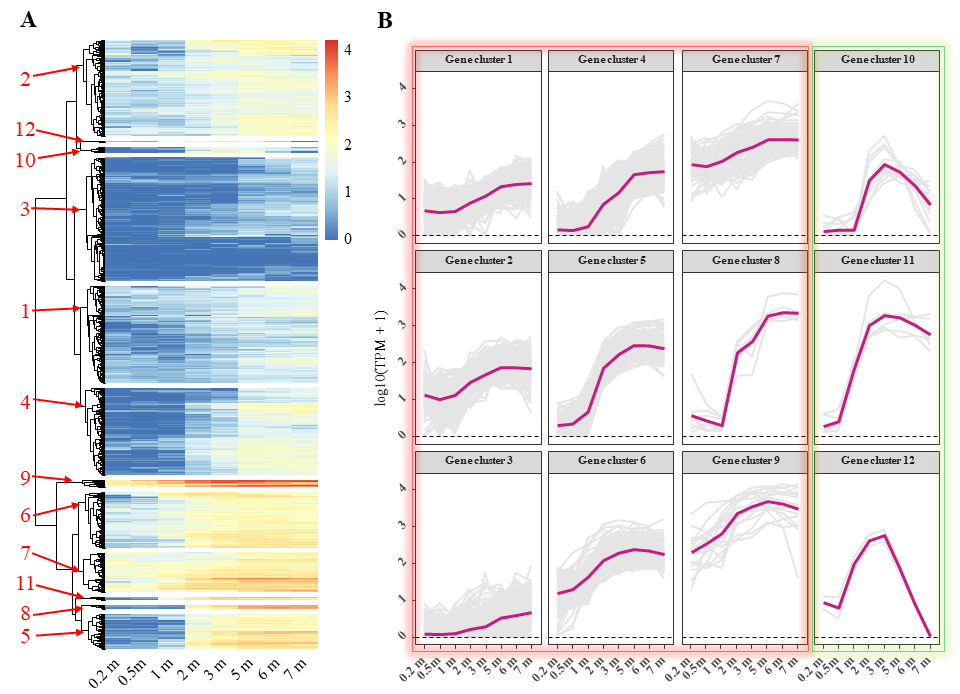


**Supplementary Fig. S3.** The expression of genes of the lncRNAs-coding gene network in moso bamboo shoots of different heights.

(A) Heatmap of genes expression profiles for log10 treatment. We classified genes into 12 clusters according to expression patterns; (B) The 12 clusters were based on expression patterns.

**Supplementary Fig. S4**


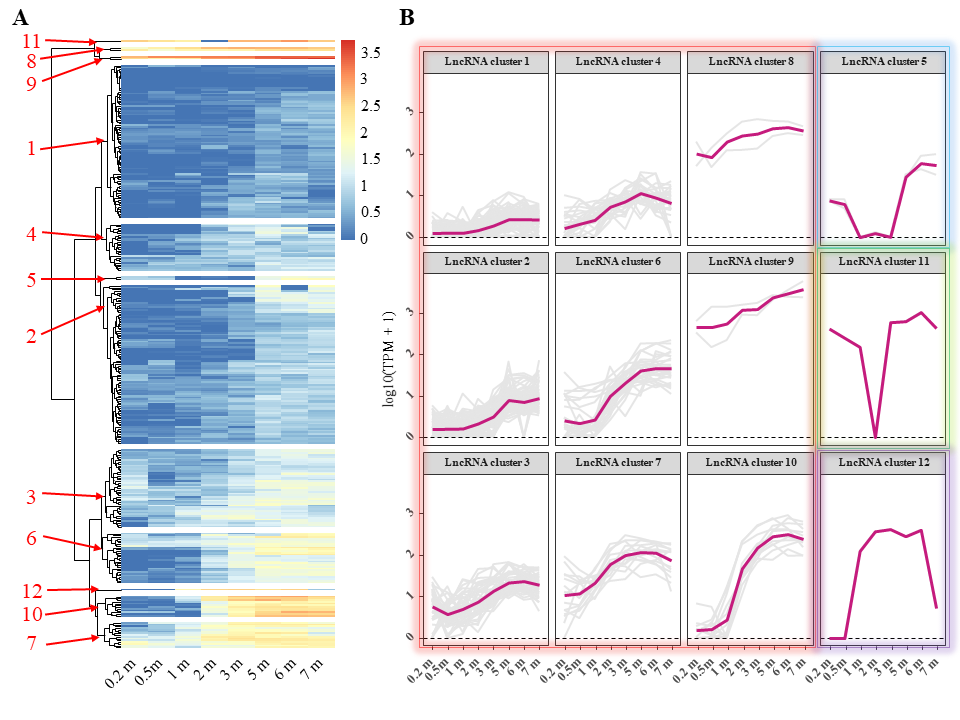


**Supplementary Fig. S4.** The expression of lncRNAs of the lncRNAs-coding gene network in moso bamboo shoots of different heights.

(A) Heatmap of the expression profile of lncRNAs forlog10 treatment. We classified lncRNAs into 12 clusters according to expression patterns; (B) The 12 clusters were based on expression patterns.

**Supplementary Fig. S5**


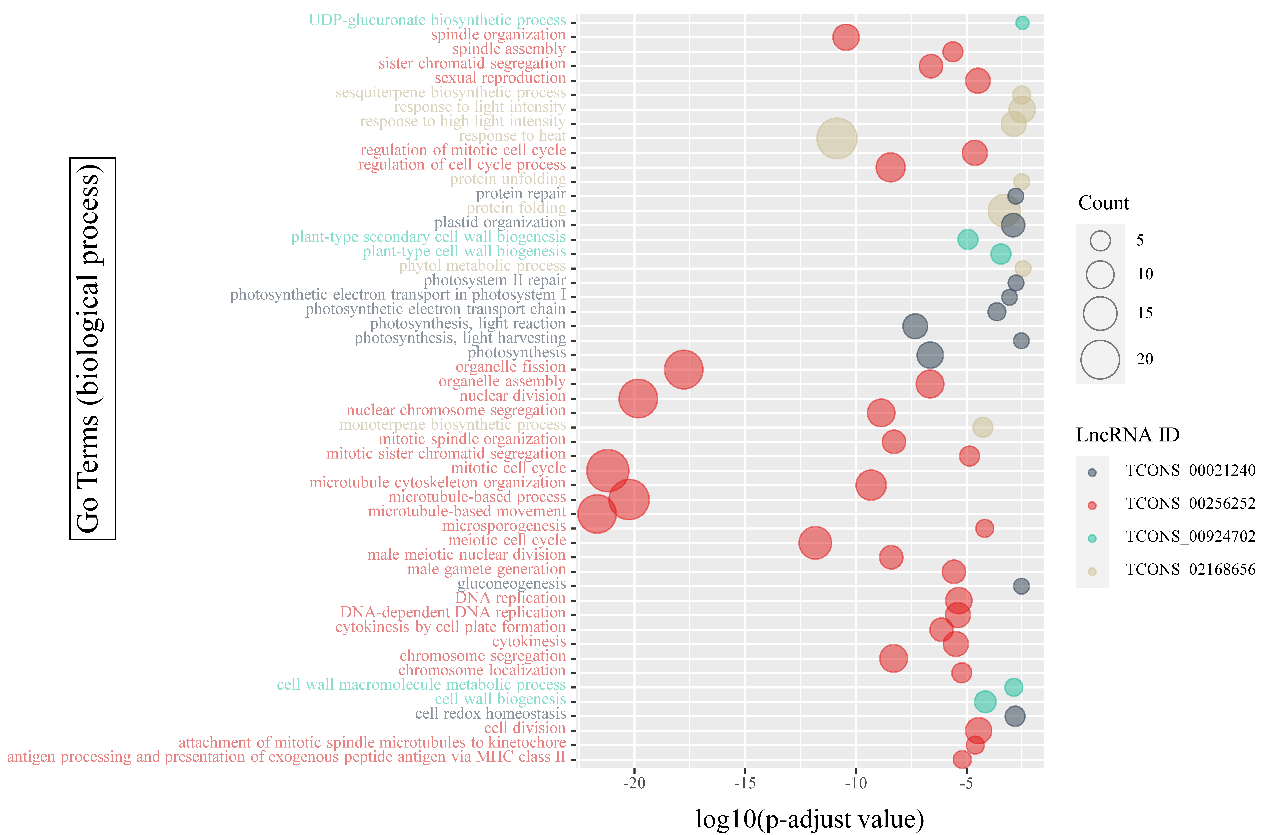


**Supplementary Fig. S5.** The enrichment annotations of lncRNAs are specifically height expressed in the rapid elongation stage.

Different colors highlighted the different lncRNAs in the GSEA analysis results. The threshold value of GSEA analysis results was p-adjust <0.05.

## Supplementary table legends

**Supplementary Table S1.** The table of RNA-Seq datasets used in this research.

A total 231 RNA-Seq datasets were used in this study. These datasets covered different tissues, stages, treatments samples.

**Supplementary Table S2.** The table of statistics in transcriptome assembly process.

RNA-Seq data were mapped to the genome of moso bamboo (released in 2018) using HISAT2, and transcripts were assembled using StringTie. Finally, we removed potential other ncRNAs, chloroplast and mitochondria sequences, to obtain clean data.

**Supplementary Table S3.** The table of samples used in tissue specific value calling.

Some samples were removed because of the deficiency of description, resulting in 148 reserved samples to annotation of tissue-specific value of lncRNAs.

**Supplementary Table S4.** The top 30 biological process terms of lncRNA functional annotation.

We calculated the number of occurrence of distinct GO terms in lncRNAs, and identified the top 30 GO terms in biological process.

**Supplementary Table S5.** The table of SCW related lncRNA list.

SCW-related lncRNAs were obtained according to the functional annotation of lncRNAs, and a total of 315 SCW-related lncRNAs were identified, along with The cluster classification and sequences information of lncRNAs.

**Supplementary Table S6.** The table of tissue-specific analysis results of 44 SCW-related lncRNAs.

The table provided the SCW-related lncRNAs for which tissue-specific annotation results were obtained. Most of lncRNAs here were specific to shoot or culm.

**Supplementary Table S7.** The lignin-related genes in lncRNA-coding gene co-expression network.

The lignin-related genes which have co-expression relationship with SCW-related lncRNAs were showed here. The orthologs of lignin-related genes were identified and provided for each gene.

**Supplementary Table S8.** The xylan-related genes in lncRNA-coding gene co-expression network.

The xylan-related genes which have co-expression relationship with SCW-related lncRNAs were showed here. The orthologs of xylan-related genes were identified and provided for each gene.

**Supplementary Table S9.** The cellulose-related genes in lncRNA-coding gene co-expression network.

The cellulose-related genes which have co-expression relationship with SCW-related lncRNAs were showed here. The orthologs of cellulose-related genes were identified and provided for each gene.

**Supplementary Table S10.** The SCW related TF genes in lncRNA-coding gene co-expression network.

The SCW-related genes which have co-expression relationship with SCW-related lncRNAs were showed here. The orthologs of SCW-related genes were identified and provided for each gene.

**Supplementary Table S11.** The table of TFs binding sites identification results.

Binding sites were called under fimo. Binding position, trend, sequences were provided.

**Supplementary Table S12.** The other TF genes in lncRNA-coding gene co-expression network.

The table shown other TF genes that have co-expression relationship with SCW-related lncRNAs in addition to SCW-related TFs. Other TF genes were annotated with orthologs identification, and the orthologs of each gene were provided.

**Supplementary Table S13.** The table of DElncRNAs list.

DElncRNAs were called using limma. The criteria were logFC > 2 and adj.P.Val < 0.001.

**Supplementary Table S14.** The table of *PAL, C4H*, and *4CL* genes in moso bamboo.

PAL, C4H, and 4CL genes in moso bamboo were obtained by orthologs identification analysis .

**Supplementary Table S15.** The GO annotations of lignin or flavonoids related co-expression genes of *PAL/C4H/4CL* genes.

Lignin- and flavonoids-related genes in *PAL/C4H/4CL* families were identified as GO annotations for their co-expressed genes. GO annotations of co-expressed genes were provided.

## Supplementary File legends

**Supplementary File F1.** The gtf file of lncRNAs identified in this study.

The file can be opened as plain file.
