# Supplementary figures and images for: Integrative lncRNA landscape reveals lncRNA-coding gene networks in the secondary cell wall biosynthesis pathway of moso bamboo (Phyllostachys edulis)
Source: BMC Genomics. 2021 Sep 4;22:638. doi: 10.1186/s12864-021-07953-z (PMC8417995; doi:10.1186/s12864-021-07953-z)

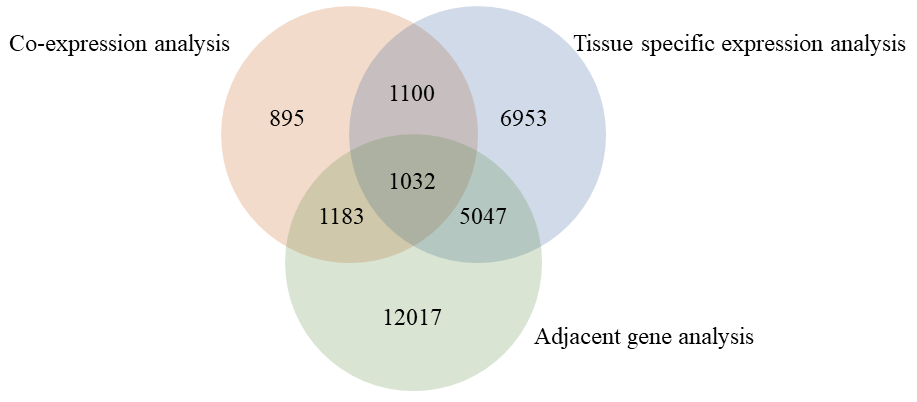

Supplement: Supplementary file 1 — Additional file 1. [file 12864_2021_7953_MOESM1_ESM.zip › Figure S1.tif]

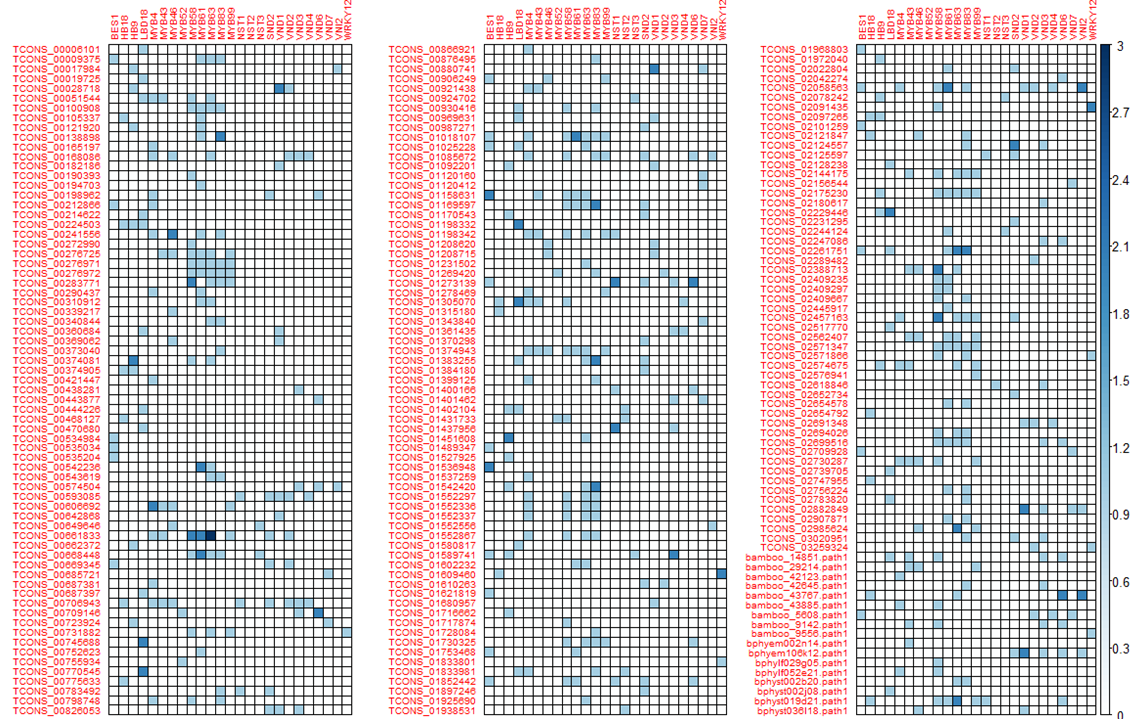

Supplement: Supplementary file 1 — Additional file 1. [file 12864_2021_7953_MOESM1_ESM.zip › Figure S2.tif]

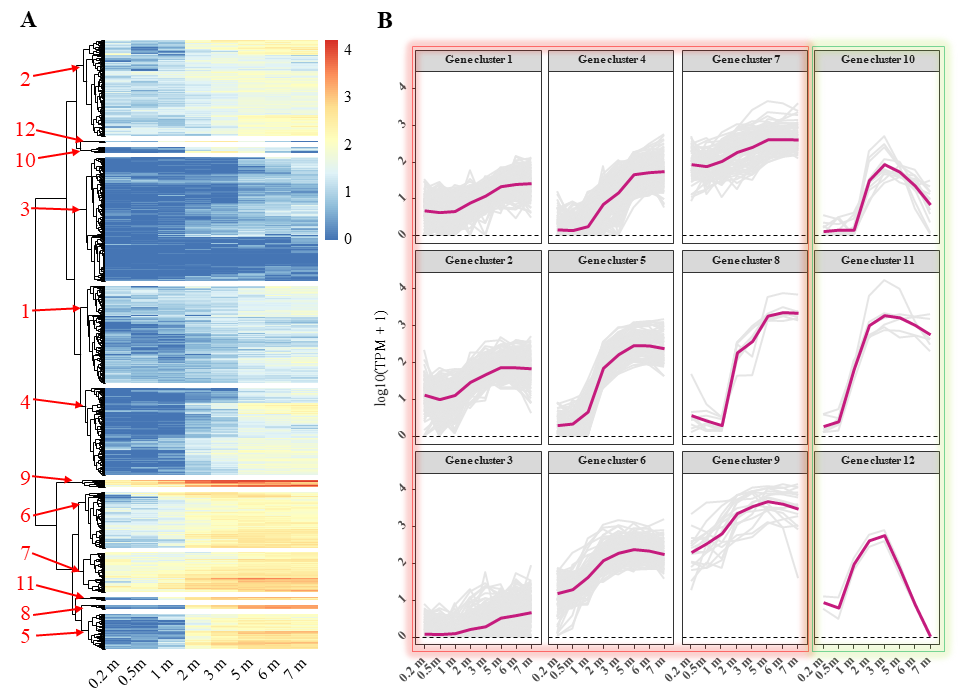

Supplement: Supplementary file 1 — Additional file 1. [file 12864_2021_7953_MOESM1_ESM.zip › Figure S3.tif]

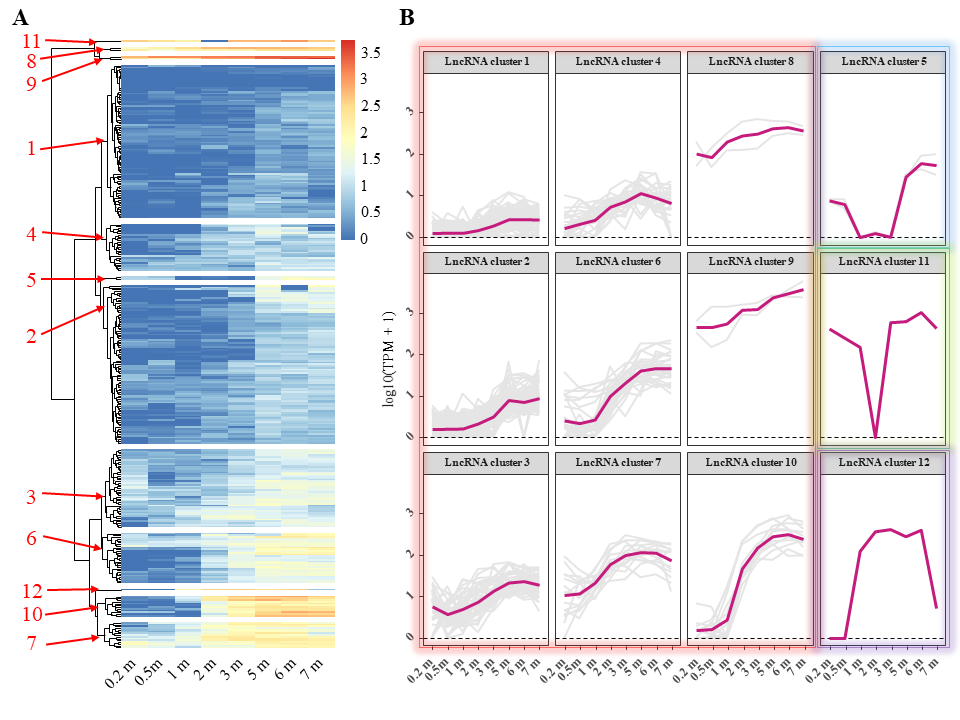

Supplement: Supplementary file 1 — Additional file 1. [file 12864_2021_7953_MOESM1_ESM.zip › Figure S4.tif]
